# Acute liver failure induced by hepatitis E virus complicated with pancreatitis and refractory acute respiratory distress syndrome after rapid ascent to high altitude: a case report and literature review

**DOI:** 10.3389/fphys.2026.1947307

**Published:** 2026-09-16

**Authors:** Bowen Wang, Mengjia Peng, Junjie Ouyang, Yangyang Liu, Youjian Hong, Jianguo Xu, Yuliang Wang

**Affiliations:** 1 Department of Emergency, General Hospital of Xizang Military Command, Lhasa, Tibet, China; 2 Intensive Care Center, General Hospital of Xizang Military Command, Lhasa, Tibet, China

**Keywords:** acute kidney injury, acute liver failure, altitude descent, hepatitis E virus, high altitude, refractory acute respiratory distress syndrome

## Abstract

**Background:**

Acute respiratory distress syndrome (ARDS) and pancreatitis are fatal extrahepatic complication of hepatitis E virus (HEV)–induced acute liver failure (ALF). High-altitude hypobaric hypoxia may exacerbate pulmonary endothelial injury and delay tissue repair, but its impact on the severity and reversibility of ALF-related pancreatitis and ARDS remain uncharacterized.

**Case presentation:**

A 44-year-old male (Body mass index 31.7 kg/m^2^) presented with jaundice, oliguria and progressive hypoxemic respiratory failure 1 month after rapid ascent from 1,190 m to 3,650 m. He was diagnosed with HEV-associated ALF, severe refractory ARDS, stage 3 acute kidney injury (AKI) and concurrent severe acute pancreatitis. Despite lung-protective ventilation, neuromuscular blockade and daily prone positioning, hypoxemia remained refractory at high altitude. The patient also received continuous renal replacement therapy and artificial liver support with dual plasma molecular adsorption system plus double filtration plasmapheresis. After 17 days, his condition stabilized but ARDS and AKI recovery was delayed. Following transfer to a hospital at 500 m altitude, oxygenation, liver and kidney function improved significantly within 3 weeks.

**Conclusion:**

Sustained high-altitude hypoxia may contribute to refractoriness and delayed recovery of ALF-related ARDS. Altitude descent may be a valuable adjuvant strategy for hemodynamically stable patients with refractory hypoxemia, pending further prospective validation.

## Introduction

Acute liver failure (ALF) caused by hepatitis E virus (HEV) is frequently complicated by extrahepatic organ dysfunction, including acute respiratory distress syndrome (ARDS), acute kidney injury (AKI), pancreatitis and coagulation dysfunction, which contribute substantially to mortality (Paul et al., 2025; Dinkelborg et al., 2026). Rapid exposure to high-altitude hypobaric hypoxia triggers systemic oxidative stress, endothelial dysfunction and exaggerated inflammatory response, and has been shown to worsen liver enzyme elevation and impair tissue repair in healthy individuals (Gatterer et al., 2024; Cai et al., 2026).

However, nearly all existing evidence focuses on primary high-altitude pulmonary edema or ARDS in otherwise healthy populations (Zhang et al., 2025). Data on the clinical course of ALF-related ARDS under high-altitude conditions are extremely scarce, and whether altitude descent can reverse refractory hypoxemia in this population remains unknown.

Here we report a case of pancreatitis and refractory severe ARDS in a patient with HEV-induced ALF after rapid ascent to 3650 m, and review relevant literature to explore the synergistic effect of hypobaric hypoxia on ALF-related pulmonary injury and the potential therapeutic role of altitude descent.

## Case presentation

A 44-year-old Han male with body mass index (BMI) of 31.7 kg/m^2^ was admitted to the intensive care unit of the General Hospital of Xizang Military Command (3,650 m, Lhasa) on May 26, 2026, with 2 days of epigastric pain and jaundice, and 1 day of progressive dyspnea and oliguria. One month prior, he had rapidly relocated from Heishui County, Sichuan (1,190 m) to Lhasa for work, with transient mild exertional dyspnea that resolved spontaneously. He had no history of chronic liver disease, cardiopulmonary disease or surgery.

Symptoms began 2 days after ingestion of spoiled food, with persistent epigastric pain, vomiting and progressive yellowing of the skin and sclera. On the day of admission, he developed generalized edema, abdominal distension and severe dyspnea. On presentation, respiratory rate was 45 breaths/min with Peripheral oxygen saturation (SpO_2_) of 68%, heart rate 134 beats/min, and blood pressure 96/63 mmHg. Physical examination showed severe icterus, coarse breath sounds with bilateral basal moist rales, abdominal distension with positive shifting dullness, positive Cullen sign and Grey-Turner sign, as well as severe pitting edema of the lower extremities.

Laboratory testing confirmed severe hyperbilirubinemia (total bilirubin 525.8 μmol/L), coagulopathy (prothrombin activity 39.7%), stage 3 acute kidney injury (serum creatinine 484.0 μmol/L), as well as markedly elevated procalcitonin (PCT) 25.89 ng/mL and C-reactive protein (CRP) 189.0 mg/L. Serum amylase (1091 U/L) and lipase (3782 U/L) were significantly elevated. Arterial blood gas on mask oxygen (fraction of inspiration O_2_, FiO_2_ 41%) showed a partial pressure of arterial oxygen (PaO_2_)/FiO_2_ ratio of 190 mmHg. Anti-HEV IgM was positive; markers for hepatitis A/B/C, HIV and syphilis were negative. Chest computed tomography (CT) revealed bilateral pulmonary infiltrates and pleural effusions; abdominal CT showed massive ascites and pancreatic enlargement with peripancreatic fat stranding (**Figure 1**). The patient was diagnosed as HEV related acute liver failure and pancreatitis (Banks et al., 2013; Shingina et al., 2023).

**Figure 1:**
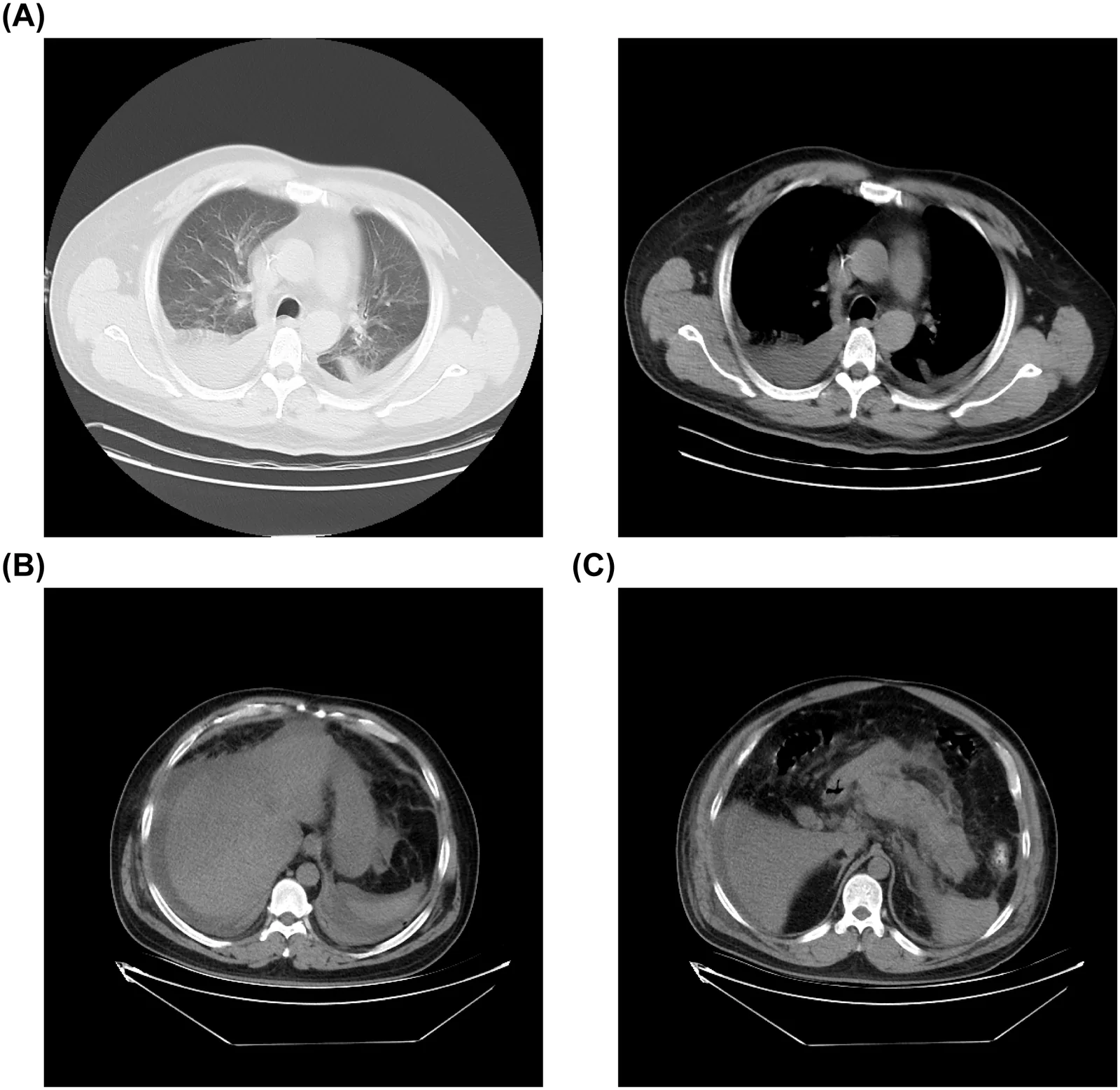
Representative computed tomography (CT) images of the patient on admission. **(A)** Chest CT scans at the thoracic level. Left panel: lung window; right panel: mediastinal window. The images demonstrate bilateral pleural effusions and pulmonary infiltrates; **(B)** Abdominal CT scan at the upper abdominal level, showing massive abdominal ascites; **(C)** Abdominal CT scan at the pancreatic level, revealing an enlarged pancreas with blurring of peripancreatic fat planes and peripancreatic inflammatory exudation, consistent with acute pancreatitis.

During 17 days of management at high altitude, the patient received comprehensive organ support therapy. For respiratory support, the patient was intubated and ventilated with lung-protective volume-assist control mode with an initial positive end-expiratory pressure (PEEP) of 10 cmH_2_O and tidal volume of 400 mL. Daily prone positioning and continuous neuromuscular blockade were initiated as adjuvant strategies for severe hypoxemia. Despite optimized ventilator settings, the PaO_2_/FiO_2_ ratio dropped below 100 mmHg on day 6 with PEEP titrated to 15 cmH_2_O, consistent with refractory ARDS (Matthay et al., 2024). On day 15, the patient was weaned from invasive ventilation and switched to high-flow nasal cannula oxygen therapy, but hypoxemia persisted (**Figure 2B**).

**Figure 2:**
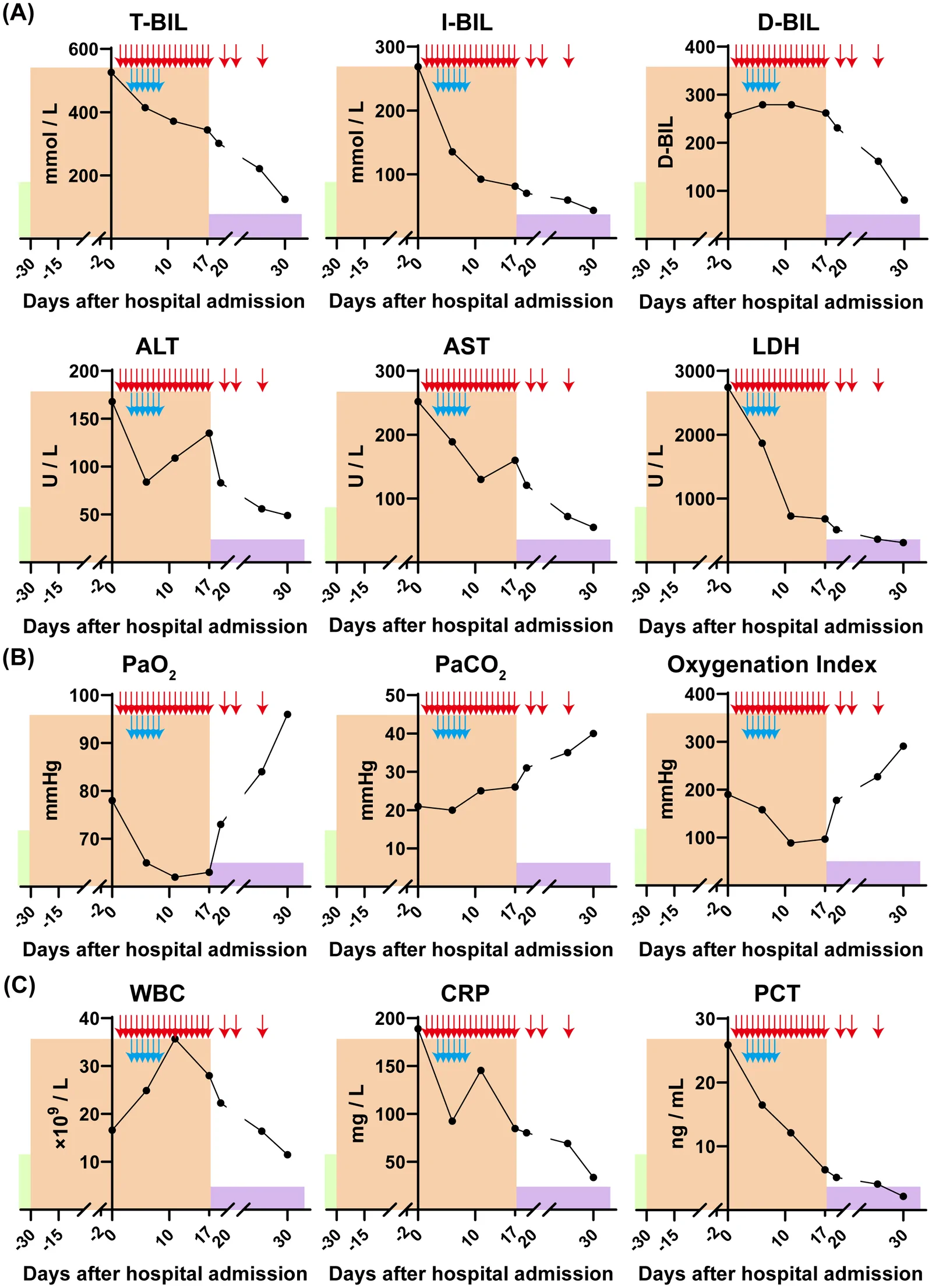
Dynamic changes in key laboratory parameters during the patient’s hospitalization. **(A)** Liver function markers: total bilirubin (T-BIL), indirect bilirubin (I-BIL), direct bilirubin (D-BIL), alanine aminotransferase (ALT), aspartate aminotransferase (AST), and lactate dehydrogenase (LDH); **(B)** Arterial blood gas parameters: arterial partial pressure of oxygen (PaO_2_), arterial partial pressure of carbon dioxide (PaCO_2_), and oxygenation index; **(C)** Inflammatory biomarkers: white blood cell count (WBC), C-reactive protein (CRP), and procalcitonin (PCT). Red arrows mark days of continuous renal replacement therapy (CRRT); blue arrows mark days of dual plasma molecular adsorption system (DPMAS) treatment. Colored shaded areas denote different altitude environments: green for Heishui County, Sichuan (1,190 m), orange for Lhasa, Tibet (3,650 m), and purple for Chengdu, Sichuan (500 m). The horizontal axis represents days after hospital admission.

For multi-organ support, six consecutive days of dual plasma molecular adsorption system (DPMAS) combined with double filtration plasmapheresis were administered starting on day 3 for progressive jaundice and coagulopathy. Continuous renal replacement therapy (CRRT) was maintained throughout the high-altitude stay to manage oliguria, AKI and electrolyte imbalance (**Figures 2A, C**) (Khwaja, 2012). Additional supportive care included broad-spectrum antibiotics, somatostatin, proton pump inhibitors for stress ulcer prophylaxis, and intravenous insulin for glycemic control.

On day 17, with stable hemodynamics but persistent delayed recovery of oxygenation and renal function (**Figures 2B**, **3A**), the patient was transferred to a tertiary hospital in Chengdu (500 m altitude). Shortly after transfer to the hospital in Chengdu, the PEEP level had been down-titrated to 6 cm H_2_O, alongside a sustained elevation in oxygenation index as illustrated in **Figure 2B**. After 3 weeks of continued supportive care, oxygenation improved markedly, liver biochemistry normalized progressively, urine volume increased (**Figures 2**, **3B**), and the patient was discharged from the intensive care unit (ICU).

**Figure 3:**
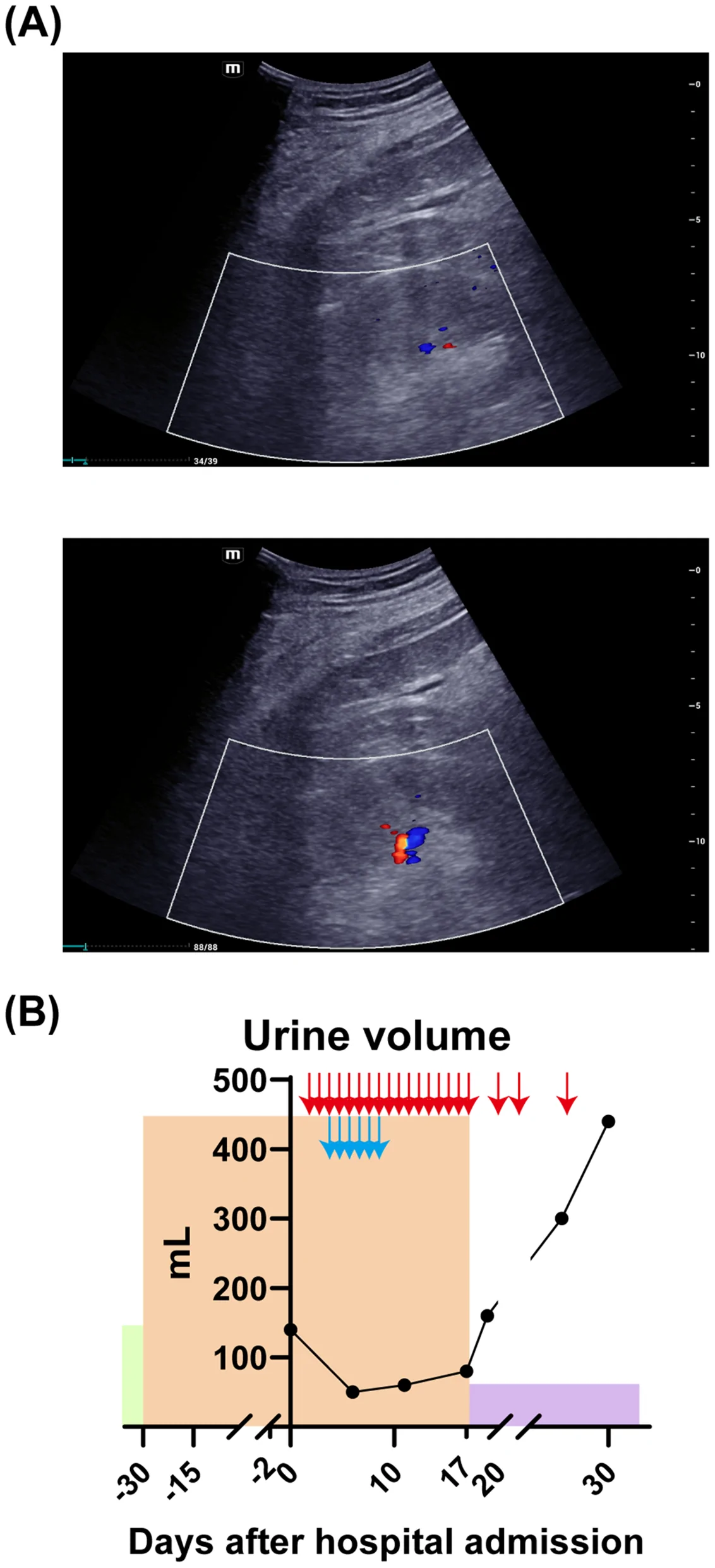
Renal color Doppler findings and dynamic changes in urine volume during the patient’s hospitalization. **(A)** Color Doppler flow imaging of the kidneys obtained on day 17 after admission (before transfer). Upper panel: left kidney; lower panel: right kidney. Scanty intrarenal cortical blood flow signals are visible in both kidneys, indicating reduced renal perfusion; No follow-up Doppler examination was performed after altitude descent. **(B)** Dynamic changes in urine volume during the patient’s hospitalization. Red arrows mark days of continuous renal replacement therapy (CRRT); blue arrows mark days of dual plasma molecular adsorption system (DPMAS) treatment. Colored shaded areas denote different altitude environments: green for Heishui County, Sichuan (1,190 m), orange for Lhasa, Tibet (3,650 m), and purple for Chengdu, Sichuan (500 m). The horizontal axis represents days after hospital admission.

## Literature review

We conducted a narrative review of published studies on HEV- induced ALF, focusing on clinical characteristics, extrahepatic complications, treatment outcomes, and existing research gaps (**Table 1**). The mortality of HEV-related ALF ranges from 2.2% to 46% across different populations and study designs. Extrahepatic complications including pancreatitis, renal dysfunction and neurological manifestations are recognized as important drivers of poor prognosis, while detailed clinical data on HEV-related pancreatitis and ARDS remain scarce. HEV infection is a globally prevalent cause of acute viral hepatitis, and a substantial proportion of severe cases progress to ALF with high mortality. Moreover, patients with pre-existing chronic liver disease or coinfection with other hepatotropic viruses are at particularly high risk of severe liver injury and fatal outcomes, highlighting the multifactorial nature of HEV- induced ALF severity (Ma et al., 2026; Sarin et al., 2026).

**Table 1:** Literature review of hepatitis E virus induced acute liver failure publications.

Author/year	Study design	Number of cases	Age	Sex	Etiology	Complications	Hospital stray	Treatment	Outcome	References
Haider Malik/2023	Case report	1	32	Male	Hepatitis A virus and Hepatitis E virus	Hepatic encephalopathy	48 hours	Dialysis	Expired	(Malik et al., 2023)
Samir Haffar/2018	Systematic review**	65	58 (median)	Male/Female	Hepatitis E virus	Hepatic encephalopathy	23 Days (median)	Liver transplantation	Expired (46%)	(Haffar et al., 2018)
Mohamed A El-Mokhtar/2023	Cohort study*	21	15 (median)	Male/Female	Hepatitis A virus and Hepatitis E virus	NR	17 Days (median)	NR	Alive	(El-Mokhtar et al., 2023)
Sayan Malakar/2024	Case report	1	72	Male	Hepatitis E virus	NR	19 Days	Prednisolone	Alive	(Malakar et al., 2024b)
Radka Komitova/2024	Cohort study*	46	65 (median)	Male/Female	Hepatitis E virus	Diabetes and cirrhosis	36 Days	Ribavirin	Expired (2.2%)	(Komitova et al., 2024)
Weixiu Li/2024	Case report	1	32	Male	Hepatitis B virus and Hepatitis E virus	Peritonitis	23 weeks	Artificial liver support system	Alive	(Li et al., 2024)
Anabella Fantilli/2021	Case report	1	56	Male	Hepatitis E virus	Hepatic encephalopathy	1 year	Liver transplantation	Expired	(Fantilli et al., 2021)
Chun Liu/2024	Case report	1	69	Male	Hepatitis E virus and SARS-CoV-2	Pneumonia	NR	Ribavirin	Alive	(Liu et al., 2024)
Francisco Josué Cordero Pérez/2024	Case report	1	54	Male	Hepatitis E virus	Pancreatitis	19 Days	Ribavirin	Alive	(Cordero Pérez et al., 2024)
Sayan Malakar/2024	Case report	1	36	Male	Hepatitis E virus	Bell’s Palsy and Pancreatitis	14 Days	Prednisolone	Alive	(Malakar et al., 2024a)
Tatsuo Kanda/2024	Case report	1	70	Male	Hepatitis E virus	Renal dysfunction	43 Days	Ribavirin	Alive	(Kanda et al., 2024)

*Cohort study; **Systematic review; NR, Not reported in the original study.

Extrahepatic organ injury is a key driver of poor prognosis in HEV and pancreatitis induced ALF, but its full spectrum remains incompletely characterized (Kamar et al., 2012; Pischke et al., 2017). Notably, although respiratory failure is recognized as a terminal event in advanced ALF, there is a striking paucity of detailed clinical data on HEV-induced ALF complicated by ARDS, particularly refractory ARDS that fails standard supportive care. This represents an important gap in the current understanding of HEV-related extrahepatic injury. In patients with established lung injury, sustained high-altitude hypoxia can convert reversible injury into refractory hypoxemia and prolong the course of ARDS (Bitos et al., 2025; Wei and Liu, 2026).

Current management of HEV- induced ALF relies largely on comprehensive supportive care, artificial liver support systems and even liver transplantation (Kamar et al., 2017). However, nearly all clinical evidence on HEV- induced ALF derives from low-altitude populations. Accumulating high-altitude medicine research indicates that sustained hypobaric hypoxia amplifies systemic inflammation, impairs hepatocyte regeneration and exacerbates pulmonary endothelial injury (Ou et al., 2013; Li et al., 2025; Zhang et al., 2025). Our case addresses this gap by describing the full clinical trajectory of HEV- induced ALF with refractory ARDS after rapid high-altitude ascent, and provides preliminary observation of the potential benefit of altitude descent.

## Discussion

This case highlights a distinct clinical phenotype: refractory severe ARDS secondary to HEV-induced ALF and pancreatitis, exacerbated by sustained exposure to 3,650 m high altitude. The most clinically relevant observation is that hypoxemia remained refractory despite maximal standard respiratory support at high altitude, but improved steadily after descent to 500 m.

The refractoriness of ARDS in this patient was explained by multiple superimposed factors. Fundamentally, HEV and pancreatitis induced ALF triggered a profound systemic inflammatory response and endothelial dysfunction, which was the core driver of pulmonary capillary leak and ARDS (Howell et al., 2026; Shen and Zhang, 2026). Concurrent severe acute pancreatitis and abdominal hypertension further reduced chest wall compliance and aggravate ventilation-perfusion mismatch (Zheng et al., 2022; Kramer et al., 2026). In addition, the patient’s class I obesity (BMI 31.7 kg/m^2^) independently decreased thoracic compliance, increased basal oxygen consumption, and elevated the risk of atelectasis and pulmonary infection, which collectively aggravated the severity of ARDS. On this basis, sustained hypobaric hypoxia at high altitude likely played a critical role in perpetuating lung injury: hypoxia amplifies pulmonary inflammation, increases vascular permeability and delays epithelial repair, creating a self-sustaining vicious cycle that might not be fully reversed by mechanical ventilation alone.

The accelerated improvement in oxygenation after altitude descent supported this pathophysiological inference. Removal of continuous hypoxia broke the cycle at its source, allowing endogenous repair mechanisms to take effect (Gatterer et al., 2024). For clinically stable patients with ALF and pancreatitis related refractory ARDS at high altitude, timely descent might therefore represent a targeted adjuvant strategy beyond conventional organ support. This case identified a transfer window that follows successful weaning to high-flow nasal cannula oxygen therapy with hemodynamic stability, which provided a practical reference for clinical decision-making.

Several important limitations must be acknowledged. First, as a single case report, causality between altitude descent and accelerated organ recovery cannot be definitively established. Multiple confounding factors may have contributed to the clinical improvement observed after transfer: (1) The natural resolution of acute pancreatitis and systemic inflammation, which corresponds to the typical recovery time course of severe acute pancreatitis at 2–3 weeks after onset; (2) Differences in medical care intensity and management strategies between the two medical institutions; (3) Delayed therapeutic effects of prior treatments including DPMAS, CRRT and anti-infective therapy, whose clinical benefits may have overlapped with the timing of transfer. Second, the patient’s obesity (BMI 31.7 kg/m2) and abdominal hypertension secondary to pancreatitis and ascites are important independent factors that increase ARDS severity and reduce chest wall compliance, and their contribution to refractory hypoxemia cannot be excluded. Third, the generalizability of this single case observation is limited. Therefore, the findings of this study only generate a clinical hypothesis and cannot be interpreted as confirmatory evidence of the therapeutic effect of altitude descent.

In conclusion, sustained high-altitude hypobaric hypoxia may contribute to the pancreatitis and refractoriness of ARDS in patients with HEV-induced ALF. Altitude descent is a reasonable therapeutic consideration for hemodynamically stable patients with persistent refractory hypoxemia. Further cohort studies are needed to quantify the benefit and define optimal transfer criteria.

## Data Availability

The original contributions presented in the study are included in the article/supplementary material. Further inquiries can be directed to the corresponding authors.
